# Hemoglobin A_1c_ Is Associated With Increased Risk of Incident Coronary Heart Disease Among Apparently Healthy, Nondiabetic Men and Women

**DOI:** 10.1161/JAHA.112.000077

**Published:** 2013-04-24

**Authors:** Jennifer K. Pai, Leah E. Cahill, Frank B. Hu, Kathryn M. Rexrode, JoAnn E. Manson, Eric B. Rimm

**Affiliations:** 1 Department of Epidemiology, Harvard School of Public Health, Boston, MA (J.K.P., F.B.H., J.A.E.M., E.B.R.); 2 Department of Nutrition, Harvard School of Public Health, Boston, MA (L.E.C., F.B.H., E.B.R.); 3 Division of Preventive Medicine, Department of Medicine, Brigham and Women's Hospital and Harvard Medical School, Boston, MA (K.M.R., J.A.E.M.); 4 Channing Division of Network Medicine, Department of Medicine, Brigham and Women's Hospital and Harvard Medical School, Boston, MA (J.K.P., F.B.H., E.B.R.)

**Keywords:** coronary heart disease, C‐reactive protein, epidemiology, glycated hemoglobin, inflammation

## Abstract

**Background:**

Hemoglobin A1c (HbA_1c_), a time‐integrated marker of glycemic control, predicts risk of coronary heart disease (CHD) among diabetics. Few studies have examined HbA_1c_ and risk of CHD among women and men without clinically elevated levels or previously diagnosed diabetes.

**Methods and Results:**

We conducted parallel nested case–control studies among women (Nurses' Health Study) and men (Health Professionals Follow‐up Study). During 14 and 10 years of follow‐up, 468 women and 454 men developed incident nonfatal myocardial infarction (MI) and fatal CHD. Controls were matched 2:1 based on age, smoking, and date of blood draw. For these analyses, participants with a history of diabetes or HbA_1c_ levels ≥6.5% at baseline were excluded. Compared with HbA_1c_ of 5.0% to <5.5%, those with an HbA_1c_ of 6.0% to <6.5% had a multivariable‐adjusted relative risk (RR) of CHD of 1.90 (95% CI 1.11 to 3.25) in women and 1.81 (95% CI 1.09 to 3.03) in men. The pooled RR of CHD was 1.29 (95% CI 1.11 to 1.50) for every 0.5%‐increment increase in HbA_1c_ levels and 1.67 (95% CI 1.23 to 2.25) for every 1%‐increment increase, with the risk plateauing around 5.0%. Furthermore, participants with HbA_1c_ levels between 6.0% and <6.5% and C‐reactive protein levels >3.0 mg/L had a 2.5‐fold higher risk of CHD compared with participants in the lowest categories of both biomarkers.

**Conclusions:**

Our findings suggest that HbA_1c_ is associated with CHD risk among apparently healthy, nondiabetic women and men and may be an important early clinical marker of disease risk.

## Introduction

Diabetes is a well‐known risk factor for cardiovascular disease (CVD),^1^ and although the exact mechanisms are unknown, angiopathy from dysglycemia is likely a primary cause. Glycated hemoglobin, assessed clinically by hemoglobin A1c (HbA_1c_), is a time‐integrated marker of average blood glucose concentration increasingly used in the screening for and management of diabetes, and it is more closely related to the risk of complications than are single or episodic measures of glucose levels.^2–4^ Despite the clinical utility of HbA_1c_, several recent large randomized trials of intensive HbA_1c_ lowering among high‐risk diabetics have not demonstrated lower risk of macrovascular events.^5–8^

Currently, the degree to which mild elevations of HbA_1c_ not in the diabetic range are associated with risk of coronary heart disease (CHD) is unclear.

The association between dysglycemia and risk of CVD may start at levels that are only modestly elevated, well below the glucose threshold for diabetes.^9–10^ The majority of studies conducted among nondiabetics have included participants with HbA_1c_ ≥6.5%,^11–12^ the threshold set for diabetes diagnosis by the American Diabetes Association^2^ and World Health Organization.^4^ In several studies, the association between HbA_1c_ and CHD is attenuated and no longer significant after adjustment for well‐known cardiovascular risk factors.^11,13^ Thus, it remains unclear whether HbA_1c_ is a clinically relevant marker of CHD risk in apparently healthy, nondiabetic populations, or if it is a proxy for dyslipidemia, hyperinsulinemia, or other metabolic pathways. In the present study, we examined the association between HbA_1c_ and risk of incident CHD in 2 prospective nested case–control studies of health professionals without diabetes or without HbA_1c_ concentrations ≥6.5%.

## Methods

### Study Population

The Nurses' Health Study (NHS) and the Health Professionals Follow‐Up Study (HPFS) are prospective cohort investigations among 121 700 female US registered nurses aged 30 to 55 years at baseline in 1976 (NHS) and 51 529 male US health professionals aged 40 to 75 years at baseline in 1986 (HPFS). Information about health and disease is assessed biennially, and information about diet is assessed every 4 years via self‐administered questionnaires.^14–15^ Between 1989 and 1990, a blood sample was requested from all participants in the NHS, and it was returned from 32 826 women. Similarly, between 1993 and 1995, a blood sample was returned from 18 225 men in the HPFS. Participants who provided blood samples were similar to those who did not, albeit somewhat younger. In the NHS, among women without CVD or cancer before 1990, we identified 468 women with incident nonfatal myocardial infarction (MI) or fatal CHD between the date of blood draw and June 2004. In the HPFS, we identified 454 men with incident nonfatal MI or fatal CHD between the date of blood draw and return of the 2004 questionnaire. Using risk‐set sampling,^16^ controls were randomly selected on a 2:1 basis matched for age, smoking, date of blood draw, and fasting status (women only) from participants free of CVD at the time the case was diagnosed. To assess men and women without diabetes, participants with a history of diabetes or HbA_1c_ levels ≥6.5% at baseline (153 women; 123 men) were excluded from further analyses. We used 6.5% as the cutoff because this value has been recommended as a level defining diabetes by the American Diabetes Association^2^ and the World Health Organization^4^ and is the level at which complications of diabetes such as retinopathy arise.

The study protocol was approved by the Institutional Review Board of the Brigham and Women's Hospital and by the Harvard School of Public Health Human Subjects Committee Review Board.

### Assessment of CHD

MI was confirmed by study physicians blinded to participants' exposure status if it met World Health Organization criteria (symptoms plus either diagnostic electrocardiographic changes or elevated levels of cardiac enzymes).^17–18^ Deaths were identified from state vital records and the National Death Index or reported by the participant's next of kin or the postal system. Fatal CHD was confirmed by use of hospital records or on autopsy or if CHD was listed as the cause of death on the death certificate, if it was the underlying and most plausible cause, and if evidence of previous CHD was available.

### Assessment of Other Factors

Anthropometric data, lifestyle behavior, and diet were derived from the questionnaire administered in 1990 to women and 1994 to men, with missing information substituted from previous questionnaires. Body mass index (BMI) was calculated as body weight divided by height squared. Average nutrient intake was computed using the semiquantitative food frequency questionnaire. Physical activity was expressed as metabolic equivalent‐hours. The questionnaires and the validity and reproducibility of measurements have been previously reported.^15,19^

### Measurement of Biochemical Variables

Blood samples were collected in liquid sodium heparin blood tubes for women and EDTA tubes for men, placed on ice packs, stored in polystyrene foam containers, returned to our laboratory via overnight courier, and centrifuged and aliquoted for storage in liquid nitrogen freezers (−130°C or colder). The first nested case–control set included cases and controls selected from 1990 to 1998 for NHS and 1994 to 2000 for HPFS and were previously documented.^20^ These samples were assayed in 2002. Since then, additional nested case–control sets with follow‐up through 2004 have been completed for both NHS and HPFS and were sent for assaying using similar methods in 2007. All analyses were performed in a laboratory certified by the Centers for Disease Control and Prevention/ National Heart, Lung, and Blood Institute Lipid Standardization Program with commercially available analytic systems.

HbA_1c_ was measured by turbidimetric immunoinhibition using packed red cells (Roche Diagnostics), which is a standard approved by the US National Glycohemoglobin Standardization Program and the Food and Drug Administration for clinical use, and traceable to the Diabetes Controls and Complications Trial. The day‐to‐day variability at HbA_1c_ values of 5.5 and 9.1 were 1.9% and 3.0%, respectively. In a substudy of 83 participants measured 3 years apart, HbA_1c_ was highly correlated between draws (Spearman=0.88) and had excellent intraclass correlations (0.73). C‐reactive protein (CRP) concentrations were determined with an immunoturbidimetric high‐sensitivity assay using reagents and calibrators from Denka Seiken with assay day‐to‐day variability between 1% and 2% and were previously shown to be largely unaffected by transport conditions and to be reproducible within persons over time.^21–22^ Total, high‐density lipoprotein (HDL), and directly assayed low‐density lipoprotein cholesterol and triglycerides were measured using standard methods with reagents from Roche Diagnostics and Genzyme. Study samples were sent to the laboratory for analysis in randomly ordered batches, and the laboratory was blinded to case–control status. The coefficients of variation for all the assays were <10%.

### Statistical Analyses

We analyzed the 2 cohorts separately. HbA_1c_ levels were categorized into quintiles based on the sex‐specific distributions among the controls as well as according to categories (<5.0%, 5.0% to <5.5%, 5.5% to <6.0%, and 6.0% to <6.5%). In the categorical analyses, the HbA_1c_ 5.0% to <5.5% category was used as the reference because there were more participants than in the <5.0% category. Because of this study design, the odds ratio derived from the logistic regression directly estimates the incidence rate (hazard) ratio and, thus, the RR.^16,23^ We analyzed the association between HbA_1c_ levels and risk of CHD using both conditional and unconditional logistic regression with adjustment for matching factors and laboratory batch. Because both analyses provided essentially the same results, we present unconditional logistic regression, which parallels the subgroup analyses. In our multivariable model, we further adjusted for parental history of MI before the age of 60 (yes/no), alcohol intake (nondrinker; 0.1 to 4.9, 5.0 to 14.9, 15.0 to 29.9, or ≥30.0 g/d; or missing), physical activity (quintiles), total cholesterol:HDL cholesterol ratio (quintiles), BMI in categories (<20, 20 to 24.9, 25 to 29.9, 30 to 34.9, and ≥35 kg/m^2^), and postmenopausal hormone therapy (yes/no, women only). Additional adjustment for hypertension (yes/no) at baseline, triglyceride levels (quintiles), and CRP levels (quintiles) was also conducted. Correlation coefficients were calculated using age‐adjusted Spearman partial correlation coefficients. To test for linear trend, we used the median HbA_1c_ levels in the control categories as a continuous variable. In addition, we conducted analyses per 0.5%‐increment increase in HbA_1c_ levels. We examined the possibly nonlinear relation between HbA_1c_ and CHD nonparametrically with restricted cubic splines.^24^ Tests for nonlinearity used the likelihood ratio test, comparing the model with only the linear term to the model with the linear and the cubic spline terms. To pool the RR estimates for men and women, we used the weighted average of estimates using the DerSimonian and Laird random effects model.^25^ Because we are using a single measure of HbA_1c_ to predict future disease over long‐term follow‐up, fluctuations in baseline biological risk factors over time may lead to an underestimation of the true association in the form of “regression dilution bias.”^26–27^ To account for potential changes in our baseline measure over time, we also present RR estimates corrected for potential regression dilution bias using the model described by Clarke et al.^28^ In brief, the β coefficients and standard errors for the entire cohort were divided by the regression dilution ratio, based on the within‐person correlation coefficient calculated from the subset of participants with repeated measurements, to obtain the corrected risk estimates for the usual relation of HbA_1c_ levels and CHD.

All *P* values presented are 2‐tailed, and *P* values <0.05 were considered statistically significant. All analyses were performed using SAS version 9 (SAS Institute).

## Results

Cardiovascular risk factors were higher or more frequent among the cases compared with the controls in both men and women (Table 1). As expected, cases were more likely to have parental history of early MI, history of hypercholesterolemia, and history of hypertension. Also, cases were more likely to have higher CRP levels and adverse lipid profiles compared with controls for both women and men. Age‐adjusted Spearman partial correlation coefficients are presented among controls for women and men (Table 2). HbA_1c_ percentage was positively correlated with CRP levels, triglycerides:HDL cholesterol ratio, triglycerides, and BMI and inversely correlated with HDL cholesterol and alcohol intake.

**Table 1.** Baseline Characteristics of Subjects With Incident Myocardial Infarction (Cases) and Matched* Event‐Free Controls Among Women (the Nurses' Health Study, 14 Years of Follow‐up) and Men (the Health Professionals Follow‐Up Study, 10 Years of Follow‐up) Without History of Diabetes and Hemoglobin A_1c_ <6.5%

Characteristics*	Nurses' Health Study			Health Professionals Follow‐Up Study		
	Cases	Controls	*P* Value*	Cases	Controls	*P* Value*
N	371	832		396	843	
Age, y	59.9±6.6	59.7±6.5	—	64.1±8.8	64.0±8.6	—
Current smokers, %	28.3	26.7	—	9.6	9.1	—
Body mass index, kg/m^2^	25.8±4.7	24.9±4.1	0.004	25.9±3.1	25.6±3.3	0.10
Hormone replacement therapy use among postmenopausal women, %	43.0	45.3	0.76	—	—	—
Parental history of myocardial infarction before age 60, %	24.5	13.9	<0.001	15.9	11.4	0.03
History of hypercholesterolemia, %	52.3	39.2	<0.001	50.0	41.2	0.004
History of hypertension, %	44.7	25.4	<0.001	34.3	28.5	0.04
NSAID use, %	67.7	69.1	0.62	46.7	43.5	0.29
Total caloric intake, kcal	1796±561	1765±510	0.37	2076±645	2044±642	0.41
Alcohol consumption, g/day	1.1 (0.0 to 6.0)	1.8 (0.0 to 8.8)	0.09	4.7 (0.9 to 15.4)	7.0 (0.9 to 18.3)	0.02
Physical activity, MET‐h/wk	12.2 (4.3 to 28.0)	12.4 (5.4 to 25.2)	0.69	22.9 (10.2 to 46.4)	26.9 (12.0 to 48.9)	0.08
HbA_1c_, %	5.5 (5.2 to 5.7)	5.4 (5.2 to 5.7)	0.01	5.6 (5.3 to 5.8)	5.5 (5.3 to 5.7)	0.01
CRP, mg/L	2.40 (1.07 to 5.44)	1.82 (0.80 to 3.80)	<0.001	1.33 (0.58 to 2.59)	0.97 (0.48 to 2.09)	<0.001
Total cholesterol, mg/dL	235±40.7	227±40.8	0.001	212±38.3	204±36.2	<0.001
HDL cholesterol, mg/dL	54.0±14.8	60.9±16.7	<0.001	42.9±11.5	46.4±12.7	<0.001
LDL cholesterol, mg/dL	146±37.0	135±37.5	<0.001	135±34.3	127±31.0	<0.001
Triglycerides, mg/dL	115 (82 to 167)	101 (74 to 141)	<0.001	139 (94 to 199)	116 (85 to 167)	<0.001
Total cholesterol:HDL cholesterol ratio	4.66±1.5	4.00±1.3	<0.001	5.19±1.3	4.66±1.3	<0.001

MET‐h indicates metabolic equivalent hours; HbA_1c_, hemoglobin A1c; CRP, C‐reactive protein; HDL, high‐density lipoprotein; LDL, low‐density lipoprotein.

* Matching criteria were age, smoking status, and date of blood draw; in women, additional matching criteria included fasting status.

* Values are mean±SD for continuous variables and proportions for categorical variables, except HbA_1c_, CRP, triglycerides, alcohol, and physical activity, which are shown as median (interquartile range).

* *P* for difference between cases and controls (unadjusted), determined by Student *t* test for rows with means, by Wilcoxon's rank sum test for rows with medians, and by χ^2^ test for rows with proportions.

**Table 2.** Age‐Adjusted Spearman Partial Correlation Coefficients Between Hemoglobin A_1c_ and Cardiovascular Risk Factors Among Controls in Women (the Nurses' Health Study) and Men (the Health Professionals Follow‐Up Study)

HbA_1c_%	NHS	HPFS
CRP	0.15^‡^	0.13^‡^
Total cholesterol	0.04	0.08*
LDL cholesterol	0.04	0.05
HDL cholesterol	−0.09^†^	−0.08*
Total cholesterol:HDL cholesterol ratio	0.09^†^	0.11^†^
Triglycerides	0.07*	0.12^‡^
BMI	0.09^†^	0.10^†^
Alcohol, g/d	−0.03	−0.08*

HbA_1c_ indicates hemoglobin A1c; NHS, Nurses' Health Study; HPFS, Health Professionals Follow‐Up Study; CRP, C‐reactive protein; LDL, low‐density lipoprotein; HDL, high‐density lipoprotein; BMI, body mass index.

*P<0.05, ^†^*P*<0.01, ^‡^*P*<0.001.

Among participants with HbA_1c_ levels <6.5% and without history of diabetes at baseline, greater HbA_1c_ levels were significantly associated with an increased risk of CHD in both women and men. Comparing extreme quintiles of HbA_1c_ levels, the multivariable‐adjusted RR of CHD was 1.70 (95% CI 1.22 to 2.36) in the pooled analysis, with a *P* for trend of <0.001 (Table 3). Because additional adjustment for history of hypertension, triglycerides, and CRP levels did not significantly attenuate the risk estimates, we refer to model 3 in multivariable‐adjusted and pooled analyses in Table 3.

**Table 3.** Estimated Relative Risks (and 95% CIs) of Myocardial Infarction During Follow‐up According to Quintiles of Baseline Percent Hemoglobin A_1c_ Among Women (Nurses' Health Study, 14 Years of Follow‐up) and Men (the Health Professionals Follow‐Up Study, 10 Years of Follow‐up)

	Quintile of HbA_1c_*					P Trend*
	1	2	3	4	5	
Nurses' Health Study (371 cases/832 controls)						
HbA_1c_, %	<5.1	5.1 to <5.3	5.3 to <5.5	5.5 to <5.7	≥5.7	
Cases/controls	62/166	61/168	75/165	84/167	89/166	
Model 1*	1.0	1.06 (0.70 to 1.62)	1.43 (0.94 to 2.18)	1.73 (1.12 to 2.66)	2.01 (1.26 to 3.21)	<0.001
Model 2*	1.0	1.06 (0.69 to 1.65)	1.39 (0.89 to 2.16)	1.59 (1.01 to 2.50)	1.78 (1.09 to 2.91)	0.009
Model 3*	1.0	1.05 (0.68 to 1.63)	1.35 (0.86 to 2.10)	1.55 (0.98 to 2.44)	1.71 (1.04 to 2.80)	0.01
Model 4*	1.0	1.05 (0.67 to 1.65)	1.39 (0.88 to 2.18)	1.52 (0.96 to 2.43)	1.70 (1.03 to 2.81)	0.02
Health Professionals Follow‐Up Study (396 cases/843 controls)						
HbA_1c_, %	<5.2	5.2 to <5.4	5.4 to <5.6	5.6 to <5.8	≥5.8	
Cases/controls	64/168	66/169	87/168	73/170	106/168	
Model 1*	1.0	1.12 (0.73 to 1.70)	1.56 (1.03 to 2.38)	1.35 (0.87 to 2.11)	1.97 (1.28 to 3.03)	0.001
Model 2*	1.0	1.10 (0.71 to 1.68)	1.52 (0.99 to 2.33)	1.19 (0.75 to 1.88)	1.67 (1.07 to 2.59)	0.03
Model 3*	1.0	1.10 (0.72 to 1.69)	1.55 (1.01 to 2.39)	1.19 (0.75 to 1.88)	1.69 (1.08 to 2.64)	0.02
Model 4*	1.0	1.08 (0.70 to 1.66)	1.57 (1.02 to 2.42)	1.17 (0.74 to 1.86)	1.69 (1.08 to 2.65)	0.02
Pooled						
Cases/controls	126/334	127/337	162/333	157/337	195/334	
Model 3*	1.0	1.08 (0.79 to 1.46)	1.45 (1.06 to 1.97)	1.36 (0.98 to 1.88)	1.70 (1.22 to 2.36)	<0.001

HbA_1c_ indicates hemoglobin A1c; NHS, Nurses' Health Study; HDL, high‐density lipoprotein; CRP, C‐reactive protein.

* Quintiles of HbA_1c_ are based on controls in each sex.

* *P* for trend based on median HbA_1c_ levels in quintiles of the control subjects.

* Adjusted for age, smoking status, date of blood drawing, fasting status (in NHS only), and laboratory batch.

* Additionally adjusted for family history of myocardial infarction before age of 60 years, alcohol intake, physical activity, nonsteroidal antiinflammatory drug use, total cholesterol:HDL cholesterol ratio, and hormone replacement therapy use among women.

* Additionally adjusted for body mass index.

* Further adjusted for hypertension and CRP.

**Figure 1.**
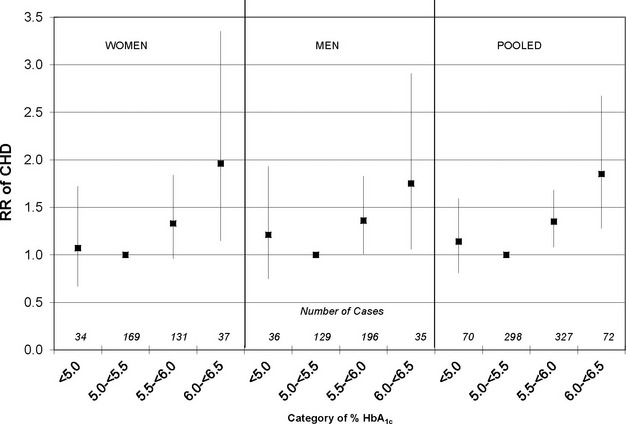
Multivariable‐adjusted* relative risk (RR) of incident CHD among women and men according to categories of HbA_1c_ levels. *Adjusted for age, smoking status, date of blood draw, fasting status (in NHS only), laboratory batch, family history of myocardial infarction before age of 60 years, alcohol intake, physical activity, NSAID use, total cholesterol:HDL cholesterol ratio, BMI, and hormone replacement therapy use among women. CHD indicates coronary heart disease; HbA_1c_, hemoglobin A1c; NHS, Nurses' Health Study; NSAID, nonsteroidal antiinflammatory drug; HDL, high‐density lipoprotein; BMI, body mass index.

**Figure 2.**
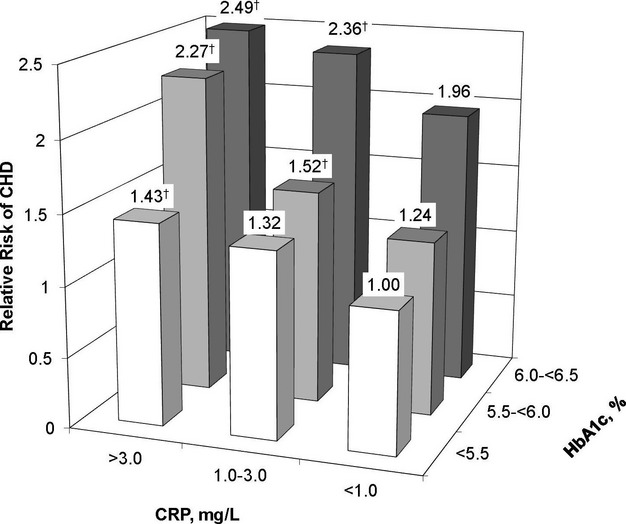
Multivariable‐adjusted* relative risk of incident CHD among women and men according to categories of HbA_1c_ and clinical cut points of CRP levels. *Adjusted for age, smoking status, date of blood draw, fasting status (in NHS only), laboratory batch, family history of myocardial infarction before age of 60 years, alcohol intake, physical activity, NSAID use, total cholesterol:HDL cholesterol ratio, BMI, and hormone replacement therapy use among women. ^†^*P*<0.05. CHD indicates coronary heart disease; HbA_1c_, hemoglobin A1c; CRP, C‐reactive protein; NHS, Nurses' Health Study; NSAID, nonsteroidal antiinflammatory drug; HDL, high‐density lipoprotein; BMI, body mass index.

We further examined the association using fixed cut points. Compared with HbA_1c_ category 5.0% to <5.5% (reference), the pooled multivariable‐adjusted RRs for CHD were 1.14 (95% CI 0.82 to 1.59) for <5.0%, 1.34 (95% CI 1.08 to 1.68) for 5.5% to <6.0%, and 1.85 (95% CI 1.28 to 2.69) for 6.0% to <6.5% (Figure 1). A significantly elevated risk of CHD was observed among nondiabetic women and men with HbA_1c_ levels of ≥5.5%. Even in these populations restricted to lower HbA_1c_ levels, we found that for every 0.5%‐increment increase in HbA_1c_ levels, the RR of CHD was 1.32 (95% CI 1.05 to 1.65) in women and 1.27 (95% CI 1.04 to 1.55) in men, and 1.29 (95% CI 1.11 to 1.50) when pooled. This association did not differ by health and lifestyle factors such as age, smoking status, BMI <25 and ≥25 kg/m^2^, history of hypertension, or parental history of early MI (Table S1). We further examined the joint effect of CRP levels based on clinical cut points for CRP and categories of HbA_1c_. Of note, in the lower risk group with CRP levels ≤3.0 mg/L, a 0.5%‐increment increase of HbA_1c_ was significantly associated with risk of incident CHD among women (RR 1.42, 95% CI 1.04 to 1.94) and men (RR 1.28, 95% CI 1.03 to 1.60). In pooled analyses, participants in the highest CRP and HbA_1c_ categories had an RR of CHD of 2.49 (95% CI 1.32 to 4.71) compared with participants in the lowest of both biomarkers (Figure 2).

A single biomarker at baseline may be a better proxy for exposure over the first several years and possibly become less correlated with exposure over a longer period. When we stratified according to the midpoint of follow‐up, the association was strongest among cases and controls in the first 7 years of follow‐up. For every 0.5%‐increment increase in HbA_1c_ levels in the earlier follow‐up (<7 years) only, the multivariable‐adjusted RR of incident CHD was 1.39 (95% CI 0.97 to 1.98) for women, 1.33 (95% CI 1.02 to 1.73) for men, and 1.35 (95% CI 1.09 to 1.67) when we pooled the estimates. In the later follow‐up (≥7 years) only, the corresponding RRs were 1.17 (95% CI 0.90 to 1.52) for women, 1.26 (95% CI 0.92 to 1.72) for men, and 1.20 (95% CI 0.98 to 1.47) when pooled. Finally, when we controlled for regression dilution bias to account for variability in the baseline measure, the pooled RR per 0.5% increment was 1.51 (95% CI 1.13 to 2.02) when restricted to the earlier follow‐up cycle and 1.42 (95% CI 1.16 to 1.74) for the entire follow‐up cycle.

## Discussion

In these prospective nested case–control studies in 2 cohorts of US health professionals, we found that HbA_1c_ was predictive of CHD risk in participants without diagnosed diabetes and with HbA_1c_ concentrations <6.5%. Men and women with an HbA_1c_ of 6.0% to <6.5% had an 85% higher risk of CHD than those with levels of 5.0% to <5.5%. The positive association persisted when adjusted for traditional cardiovascular risk factors, including hyperlipidemia, anthropometric variables, and CRP. Our results suggest that HbA_1c_ is an important marker of risk across a broad range of levels and that the causal etiologic pathway may be complex and include mechanisms other than lipids or inflammation.

The relationship of HbA_1c_ with CHD has been studied in several other nondiabetic populations^11–12,11–32^ with inconsistent results, possibly due to diverse study protocols, a wide range of length of follow‐up, inconsistent exclusion criteria, and differences in cohort demographics and outcome definitions. Two previous studies in particular have examined HbA_1c_ in relation to risk of CHD in participants without diabetes or clinically elevated HbA_1c_ levels.^13,29^ After 7 years of follow‐up in a nested case–control analysis from the WHS, Blake et al reported an RR of 2.25 (95% CI 1.59 to 3.19) for CVD events (368 cases of incident MI, stroke, or coronary revascularization) comparing the highest (≥5.50%) with the lowest (<5.23%) quartile of HbA_1c_.^13^ However, this association was attenuated (RR 1.00; 95% CI 0.65 to 1.54) after adjustment for BMI, blood pressure, CRP, low‐density lipoprotein and HDL cholesterol, and triglycerides.^13^ The authors note that the same results were observed when the end point of CHD was examined alone, although with limited power because the CHD analysis was limited to 136 cases. In a recent analysis from the Atherosclerosis Risk in Communities (ARIC) study, the risk factor–adjusted hazard ratios for CHD in participants with HbA_1c_ of 5.5% to <6.0% and of 6.0% to <6.5% were 1.23 (95% CI 1.07 to 1.41) and 1.78 (95% CI 1.48 to 2.15), respectively, compared with participants with baseline HbA_1c_ concentrations of 5.0% to <5.5%.^29^ Our results were similar in magnitude to this study. Furthermore, we showed a significant joint effect of mildly elevated HbA_1c_ and CRP levels on the risk of incident CHD among nondiabetic men and women.

Interestingly, we observed a joint association similar to that of Sander et al,^33^ who reported that among nondiabetics, the combination of elevated HbA_1c_ and CRP was associated with advanced early carotid atherosclerosis progression and a 3‐fold increased risk of major vascular events. This association between elevated HbA_1c_ and early atherosclerosis progression supports the hypothesis that HbA_1c_ may be causally related to CHD even at low levels.^33^ Hyperglycemia, in addition to diabetes, can lead to the formation of advanced glycosylated end products, which have been shown to induce production and secretion of inflammatory cytokines.^34^ Indeed, HbA_1c_ has been reported to predict CRP in established diabetes.^35^ In addition, nitric oxide bioavailability, a marker of intimal reactivity, has been shown to be reduced by both CRP^36^ and HbA_1c_.^37^ This joint effect was observed at even mildly elevated levels of both biomarkers and may influence CHD risk through mechanisms other than diabetes, atherosclerosis, and inflammation.

A recent meta‐analysis^38^ examined the association between HbA_1c_ and risk of CHD in persons without diabetes, although most studies included participants with HbA_1c_ well beyond the established clinical threshold of 6.5%. Using data from 9 cohorts (1639 cases), the meta‐analysis reported an RR of 1.20 (95% Cl 1.10‐1.31) for each 1%‐increment increase in levels of HbA_1c_.^38^ This risk estimate was not adjusted for established CHD risk factors and was weaker than our own. These differences may be due to the inherent weakness of meta‐analyses, where pooling results from many studies may lead to random error from differences in lab methods, study design, and residual confounding. In our nested prospective studies, the pooled RR of CHD for men and women was 1.67 (95% CI 1.23 to 2.25) per 1%‐increment increase in HbA_1c_, even after detailed adjustment for other risk factors.

In the present study, even a 0.5%‐increment increase of HbA_1c_ was significantly associated with risk of incident CHD among both women and men. Although a 0.5%‐increment decrease in HbA_1c_ in patients undergoing treatment for diabetes^39^ and morbid obesity^40^ has proven health benefits, there are no published experimental trials that show a similar decrease in HbA_1c_ leads to a reduction in CHD or CVD end points in the general population. Nevertheless, the most effective ways of reducing HbA_1c_, namely dietary changes, increased physical activity, and weight loss, have been shown to have beneficial effects on other cardiovascular risk factors. HbA_1c_ in nondiabetic individuals may be successfully reduced through nonpharmacologic lifestyle interventions, as demonstrated in the Finnish Diabetes Prevention Study,^41–42^ Diabetes Prevention Program^43^ and Outcomes Study,^44^ and Look AHEAD study.^45^ In a small intervention study of nondiabetic obese males, the mean HbA_1c_ levels were significantly lower after the weight loss intervention compared with the before‐intervention levels (4.9% versus 5.1%),^46^ and in a randomized controlled trial of male construction workers, lifestyle intervention aimed at energy balance was significantly associated with improved cardiovascular risk factors, including body weight, blood pressure, and lipid and HbA_1c_ levels.^47^ Although whether lowering HbA_1c_ lowers risk of future CHD among nondiabetic individuals has not been directly tested, working to optimize cardiovascular health in individuals with mildly higher HbA_1c_ seems prudent and clinically important.

Strengths of the present analysis include the long duration of follow‐up, the prospective nature of the data collection, replication in a second cohort, and comprehensive lifestyle and health factor data used for detailed multivariable adjustment. A few limitations should be addressed. First, the potential exists for measurement error in lifestyle data ascertained from self‐reported questionnaires. However, the reproducibility and validity of the self‐reported questionnaire have been well‐documented in these 2 populations of health professionals.^15,48^ Second, our study was conducted in 2 cohorts of middle‐aged, predominantly white health professionals and may not be generalizeable to other populations. However, the relationship between HbA_1c_ and CHD risk was similar in a recent community‐based study among whites and African Americans.^29^ Third, we only had a single measurement of HbA_1c_ at baseline and did not have glucose measurements. However, one advantage of using HbA_1c_ is that it is stable in stored samples and reflects glycemic control over an average of 3 months rather than measures such as glucose, which are susceptible to daily fluctuations.^49^ In addition, we observed excellent within‐person reproducibility in repeated samples measured 3 years apart and conducted additional analyses correcting for potential changes in the baseline measurement over time.

In summary, we found that HbA_1c_ is a predictor of CHD risk among apparently healthy women and men without diabetes and with HbA_1c_ <6.5%. Further examination is warranted to determine whether reducing HbA_1c_ among otherwise intermediate‐ and low‐risk individuals without diagnosed diabetes will prevent incident CHD events.

## References

[1] Sarwar N Gao P Seshasai SR Gobin R Kaptoge S Di Angelantonio E Ingelsson E Lawlor DA Selvin E Stampfer M Stehouwer CD Lewington S Pennells L Thompson A Sattar N White IR Ray KK Danesh J Diabetes mellitus, fasting blood glucose concentration, and risk of vascular disease: a collaborative meta‐analysis of 102 prospective studies. Lancet. 2010; 375:2215-2222 20609967 10.1016/S0140-6736(10)60484-9 PMC2904878

[2] American Diabetes Association Diagnosis and classification of diabetes mellitus. Diabetes Care. 2012; 35suppl 1:S64-S71 22187472 10.2337/dc12-s064 PMC3632174

[3] Gerstein HC Glycosylated hemoglobin: finally ready for prime time as a cardiovascular risk factor. Ann Intern Med. 2004; 141:475-476 15381522 10.7326/0003-4819-141-6-200409210-00014

[4] World Health Organization Use of Glycated Haemoglobin (HbA1c) in the Diagnosis of Diabetes Mellitus. Whox002Fnmhx002Fchpx002Fcpmx002F11.1. Geneva, Switzerland: World Health Organization; 2011:25

[5] Intensive blood‐glucose control with sulphonylureas or insulin compared with conventional treatment and risk of complications in patients with type 2 diabetes (UKPDS 33). UK Prospective Diabetes Study (UKPDS) group. Lancet. 1998; 352:837-853 9742976

[6] Gerstein HC Miller ME Byington RP Goff DC Jr Bigger JT Buse JB Cushman WC Genuth S Ismail‐Beigi F Grimm RH Jr Probstfield JL Simons‐Morton DG Friedewald WT Effects of intensive glucose lowering in type 2 diabetes. N Engl J Med. 2008; 358:2545-2559 18539917 10.1056/NEJMoa0802743 PMC4551392

[7] Patel A MacMahon S Chalmers J Neal B Billot L Woodward M Marre M Cooper M Glasziou P Grobbee D Hamet P Harrap S Heller S Liu L Mancia G Mogensen CE Pan C Poulter N Rodgers A Williams B Bompoint S de Galan BE Joshi R Travert F Intensive blood glucose control and vascular outcomes in patients with type 2 diabetes. N Engl J Med. 2008; 358:2560-2572 18539916 10.1056/NEJMoa0802987

[8] Duckworth W Abraira C Moritz T Reda D Emanuele N Reaven PD Zieve FJ Marks J Davis SN Hayward R Warren SR Goldman S McCarren M Vitek ME Henderson WG Huang GD Glucose control and vascular complications in veterans with type 2 diabetes. N Engl J Med. 2009; 360:129-139 19092145 10.1056/NEJMoa0808431

[9] Gerstein HC More insights on the dysglycaemia‐cardiovascular connection. Lancet. 2010; 375:2195-2196 20609955 10.1016/S0140-6736(10)60973-7

[10] Gerstein HC Yusuf S Dysglycaemia and risk of cardiovascular disease. Lancet. 1996; 347:949-950 8598762 10.1016/s0140-6736(96)91420-8

[11] Selvin E Coresh J Golden SH Brancati FL Folsom AR Steffes MW Glycemic control and coronary heart disease risk in persons with and without diabetes: the Atherosclerosis Risk in Communities Study. Arch Intern Med. 2005; 165:1910-1916 16157837 10.1001/archinte.165.16.1910

[12] Khaw KT Wareham N Bingham S Luben R Welch A Day N Association of hemoglobin A1c with cardiovascular disease and mortality in adults: the European prospective investigation into cancer in Norfolk. Ann Intern Med. 2004; 141:413-420 15381514 10.7326/0003-4819-141-6-200409210-00006

[13] Blake GJ Pradhan AD Manson JE Williams GR Buring J Ridker PM Glynn RJ Hemoglobin A1c level and future cardiovascular events among women. Arch Intern Med. 2004; 164:757-761 15078645 10.1001/archinte.164.7.757

[14] Colditz GA The Nurses' Health Study: a cohort of US women followed since 1976. J Am Med Womens Assoc. 1995; 50:40-44 7722205

[15] Rimm EB Giovannucci EL Stampfer MJ Colditz GA Litin LB Willett WC Reproducibility and validity of an expanded self‐administered semiquantitative food frequency questionnaire among male health professionals. Am J Epidemiol. 1992; 135:1114-1126 1632423 10.1093/oxfordjournals.aje.a116211

[16] Prentice RL Breslow NE Retrospective studies and failure time models. Biometrika. 1978; 65:153-158

[17] Luepker RV Apple FS Christenson RH Crow RS Fortmann SP Goff D Goldberg RJ Hand MM Jaffe AS Julian DG Levy D Manolio T Mendis S Mensah G Pajak A Prineas RJ Reddy KS Roger VL Rosamond WD Shahar E Sharrett AR Sorlie P Tunstall‐Pedoe H Case definitions for acute coronary heart disease in epidemiology and clinical research studies: a statement from the AHA Council on Epidemiology and Prevention; AHA Statistics Committee; World Heart Federation Council on Epidemiology and Prevention; the European Society of Cardiology Working Group on Epidemiology and Prevention; Centers for Disease Control and Prevention; and the National Heart, Lung, and Blood Institute. Circulation. 2003; 108:2543-2549 14610011 10.1161/01.CIR.0000100560.46946.EA

[18] Rose GA, Blackburn H. Cardiovascular Survey Methods. WHO monograph series no. 58. Geneva, Switzerland: World Health Organization; 1982 4972212

[19] Willett W Lenart E In: Willett W (ed.). Reproducibility and validity of food‐frequency questionnaires. Nutritional Epidemiology. 1998 New York, NY Oxford University Press 101-147

[20] Pai JK Pischon T Ma J Manson JE Hankinson SE Joshipura K Curhan GC Rifai N Cannuscio CC Stampfer MJ Rimm EB Inflammatory markers and the risk of coronary heart disease in men and women. N Engl J Med. 2004; 351:2599-2610 15602020 10.1056/NEJMoa040967

[21] Pai JK Curhan GC Cannuscio CC Rifai N Ridker PM Rimm EB Stability of novel plasma markers associated with cardiovascular disease: processing within 36 hours of specimen collection. Clin Chem. 2002; 48:1781-1784 12324497

[22] Pischon T Hankinson SE Hotamisligil GS Rifai N Willett WC Rimm EB Habitual dietary intake of n‐3 and n‐6 fatty acids in relation to inflammatory markers among US men and women. Circulation. 2003; 108:155-160 12821543 10.1161/01.CIR.0000079224.46084.C2

[23] Rothman KJ Greenland S In: Rothman KJ Greenland S (eds.). Case‐control studies. Modern Epidemiology. 1998 Philadelphia, Pa Lippincott‐Raven Publishers 93-114

[24] Durrleman S Simon R Flexible regression models with cubic splines. Stat Med. 1989; 8:551-561 2657958 10.1002/sim.4780080504

[25] Takkouche B Cadarso‐Suarez C Spiegelman D Evaluation of old and new tests of heterogeneity in epidemiologic meta‐analysis. Am J Epidemiol. 1999; 150:206-215 10412966 10.1093/oxfordjournals.aje.a009981

[26] Lewington S Whitlock G Clarke R Sherliker P Emberson J Halsey J Qizilbash N Peto R Collins R Blood cholesterol and vascular mortality by age, sex, and blood pressure: a meta‐analysis of individual data from 61 prospective studies with 55,000 vascular deaths. Lancet. 2007; 370:1829-1839 18061058 10.1016/S0140-6736(07)61778-4

[27] Wu JH Lemaitre RN King IB Song X Sacks FM Rimm EB Heckbert SR Siscovick DS Mozaffarian D Association of plasma phospholipid long‐chain omega‐3 fatty acids with incident atrial fibrillation in older adults: the Cardiovascular Health Study. Circulation. 2012; 125:1084-1093 22282329 10.1161/CIRCULATIONAHA.111.062653 PMC3302663

[28] Clarke R Shipley M Lewington S Youngman L Collins R Marmot M Peto R Underestimation of risk associations due to regression dilution in long‐term follow‐up of prospective studies. Am J Epidemiol. 1999; 150:341-353 10453810 10.1093/oxfordjournals.aje.a010013

[29] Selvin E Steffes MW Zhu H Matsushita K Wagenknecht L Pankow J Coresh J Brancati FL Glycated hemoglobin, diabetes, and cardiovascular risk in nondiabetic adults. N Engl J Med. 2010; 362:800-811 20200384 10.1056/NEJMoa0908359 PMC2872990

[30] Pradhan AD Rifai N Buring JE Ridker PM Hemoglobin A1c predicts diabetes but not cardiovascular disease in nondiabetic women. Am J Med. 2007; 120:720-727 17679132 10.1016/j.amjmed.2007.03.022 PMC2585540

[31] Adams RJ Appleton SL Hill CL Wilson DH Taylor AW Chittleborough CR Gill TK Ruffin RE Independent association of HbA(1c) and incident cardiovascular disease in people without diabetes. Obesity (Silver Spring). 2009; 17:559-563 19131940 10.1038/oby.2008.592

[32] Lawlor DA Fraser A Ebrahim S Smith GD Independent associations of fasting insulin, glucose, and glycated haemoglobin with stroke and coronary heart disease in older women. PLoS Med. 2007; 4:e263 17760500 10.1371/journal.pmed.0040263 PMC1952205

[33] Sander D Schulze‐Horn C Bickel H Gnahn H Bartels E Conrad B Combined effects of hemoglobin A1c and C‐reactive protein on the progression of subclinical carotid atherosclerosis: the INVADE study. Stroke. 2006; 37:351-357 16373634 10.1161/01.STR.0000199034.26345.bc

[34] Vlassara H Brownlee M Manogue KR Dinarello CA Pasagian A Cachectin/TNF and IL‐1 induced by glucose‐modified proteins: role in normal tissue remodeling. Science. 1988; 240:1546-1548 3259727 10.1126/science.3259727

[35] King DE Mainous AG III Buchanan TA Pearson WS C‐reactive protein and glycemic control in adults with diabetes. Diabetes Care. 2003; 26:1535-1539 12716818 10.2337/diacare.26.5.1535

[36] Verma S Wang CH Li SH Dumont AS Fedak PW Badiwala MV Dhillon B Weisel RD Li RK Mickle DA Stewart DJ A self‐fulfilling prophecy: C‐reactive protein attenuates nitric oxide production and inhibits angiogenesis. Circulation. 2002; 106:913-919 12186793 10.1161/01.cir.0000029802.88087.5e

[37] Giugliano D Marfella R Coppola L Verrazzo G Acampora R Giunta R Nappo F Lucarelli C D'Onofrio F Vascular effects of acute hyperglycemia in humans are reversed by l‐arginine. Evidence for reduced availability of nitric oxide during hyperglycemia. Circulation. 1997; 95:1783-1790 9107164 10.1161/01.cir.95.7.1783

[38] Sarwar N Aspelund T Eiriksdottir G Gobin R Seshasai SR Forouhi NG Sigurdsson G Danesh J Gudnason V Markers of dysglycaemia and risk of coronary heart disease in people without diabetes: Reykjavik prospective study and systematic review. PLoS Med. 2010; 7:e1000278 20520805 10.1371/journal.pmed.1000278 PMC2876150

[39] Umpierre D Ribeiro PA Kramer CK Leitao CB Zucatti AT Azevedo MJ Gross JL Ribeiro JP Schaan BD Physical activity advice only or structured exercise training and association with HbA1c levels in type 2 diabetes: a systematic review and meta‐analysis. JAMA. 2011; 305:1790-1799 21540423 10.1001/jama.2011.576

[40] Li Q Chen L Yang Z Ye Z Huang Y He M Zhang S Feng X Gong W Zhang Z Zhao W Liu C Qu S Hu R Metabolic effects of bariatric surgery in type 2 diabetic patients with body mass index <35 kg/m^2^. Diabetes Obes Metab. 2011; 14:262-270 22051116 10.1111/j.1463-1326.2011.01524.x

[41] Tuomilehto J Lindstrom J Eriksson JG Valle TT Hamalainen H Ilanne‐Parikka P Keinanen‐Kiukaanniemi S Laakso M Louheranta A Rastas M Salminen V Uusitupa M Prevention of type 2 diabetes mellitus by changes in lifestyle among subjects with impaired glucose tolerance. N Engl J Med. 2001; 344:1343-1350 11333990 10.1056/NEJM200105033441801

[42] Lindstrom J Ilanne‐Parikka P Peltonen M Aunola S Eriksson JG Hemio K Hamalainen H Harkonen P Keinanen‐Kiukaanniemi S Laakso M Louheranta A Mannelin M Paturi M Sundvall J Valle TT Uusitupa M Tuomilehto J Sustained reduction in the incidence of type 2 diabetes by lifestyle intervention: follow‐up of the Finnish Diabetes Prevention Study. Lancet. 2006; 368:1673-1679 17098085 10.1016/S0140-6736(06)69701-8

[43] Knowler WC Barrett‐Connor E Fowler SE Hamman RF Lachin JM Walker EA Nathan DM Reduction in the incidence of type 2 diabetes with lifestyle intervention or metformin. N Engl J Med. 2002; 346:393-403 11832527 10.1056/NEJMoa012512 PMC1370926

[44] Perreault L Pan Q Mather KJ Watson KE Hamman RF Kahn SE Effect of regression from prediabetes to normal glucose regulation on long‐term reduction in diabetes risk: results from the Diabetes Prevention Program Outcomes Study. Lancet. 2012; 379:2243-2251 22683134 10.1016/S0140-6736(12)60525-X PMC3555407

[45] Wing RR Lang W Wadden TA Safford M Knowler WC Bertoni AG Hill JO Brancati FL Peters A Wagenknecht L Benefits of modest weight loss in improving cardiovascular risk factors in overweight and obese individuals with type 2 diabetes. Diabetes Care. 2011; 34:1481-1486 21593294 10.2337/dc10-2415 PMC3120182

[46] Kotani K Yamada T Taniguchi N The association between circulating secreted protein acidic and rich in cysteine (SPARC) and glycosylated haemoglobin (HbA(1c)) during lifestyle‐modified weight reduction intervention in obese male subjects. J Int Med Res. 2011; 39:528-532 21672357 10.1177/147323001103900221

[47] Groeneveld IF Proper KI van der Beek AJ van Mechelen W Sustained body weight reduction by an individual‐based lifestyle intervention for workers in the construction industry at risk for cardiovascular disease: results of a randomized controlled trial. Prev Med. 2010; 51:240-246 20692282 10.1016/j.ypmed.2010.07.021

[48] Feskanich D Rimm EB Giovannucci EL Colditz GA Stampfer MJ Litin LB Willett WC Reproducibility and validity of food intake measurements from a semiquantitative food frequency questionnaire. J Am Diet Assoc. 1993; 93:790-796 8320406 10.1016/0002-8223(93)91754-e

[49] Little RR Rohlfing CL Tennill AL Connolly S Hanson S Effects of sample storage conditions on glycated hemoglobin measurement: evaluation of five different high performance liquid chromatography methods. Diabetes Technol Ther. 2007; 9:36-42 17316096 10.1089/dia.2006.0055

